# Variations in right atrial flow patterns in the normal heart a potential contributor to cryptogenic stroke in the setting of patent foramen ovale

**DOI:** 10.1186/1532-429X-17-S1-P28

**Published:** 2015-02-03

**Authors:** Jehill D Parikh, Jayant Kakarla, Kieren G Hollingsworth, Bernard Keavney, John J O'Sullivan, Gary A Ford, Andrew M Blamire, Louise Coats

**Affiliations:** 1 Institute of Genetic Medicine, Newcastle University, Newcastle upon Tyne, UK; 2 Department of Congenital Cardiology, Freeman Hospital, Newcastle upon Tyne, UK; 3 Institute of Cellular Medicine, Newcastle University, Newcastle upon Tyne, UK; 4 Institute of Cardiovascular Sciences, University of Manchester, Manchester, UK; 5 Medical Sciences Division, University of Oxford, Oxford, UK

## Background

Patent foramen ovale (PFO) occurs in a quarter of the population but half of those with cryptogenic stroke (CS). Difficulty in identifying the pathogenic PFO versus the innocent bystander has contributed to controversy surrounding outcomes following PFO closure. We aimed to investigate whether right atrial flow patterns could help define the mechanism for CS in the setting of PFO.

## Methods

4D flow cardiac magnetic resonance (CMR) was performed in 10 subjects (age 43±6 years, 4 male) with proven PFO and CS and in 10 controls (age 42±7years, 4 male) with normal trans-thoracic echocardiograms. CMR was performed at 3T (Achieva; Philips) with a 6-channel cardiac array. A retrospectively ECG-gated and respiratory-gated TFE sequence (TE/TR/flip:3.7/6.3ms/8°, VENC:150m/s, FOV:240mm(AP)x240mm(FH)x142mm(LR), spatial resolution:3mm^3^, temporal resolution:50-55ms, 20 phases, SENSE, factor 2) was used. Analysis was performed with GTflow v2.0 (Gyrotools). Contours were placed manually in the superior vena cava (SVC) and inferior vena cava (IVC) in the axial plane at the junction with the right atrium. Net flow was assessed at these points. The relative position of the SVC and IVC at these points was measured. A pathline analysis of atrial flow was performed (SVC:red, IVC:green).

## Results

**Figure 1:**
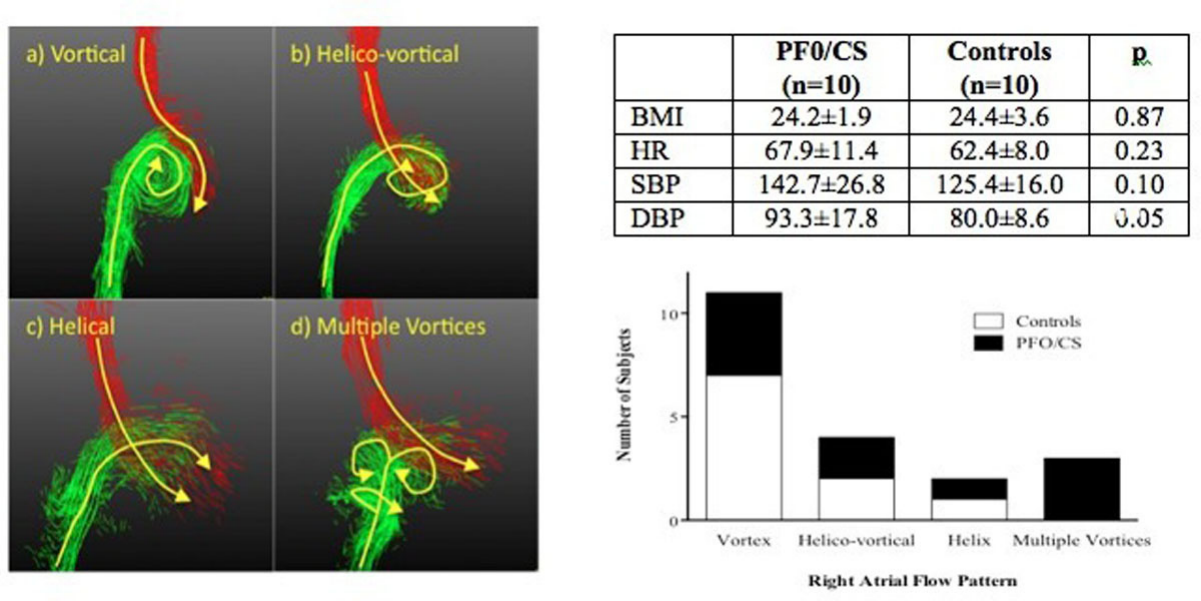


Body mass index (BMI) and heart rate (HR) were comparable but blood pressure was higher in the PFO/CS group with three patients taking anti-hypertensives (Table). Four variations of right atrial flow were observed (Figure 1):

a) vortical [clockwise vortex, conventionally described]

b) helico-vortical [IVC forms vortex, SVC passes laterally enveloping in a helical fashion]

c) helical [SVC and IVC flow twist together in a helix]

d) multiple vortices [arising variably from SVC and IVC flow]

Vortical flow was most prevalent in the control group whilst the PFO/CS subjects were more likely to show one of the other flow patterns (Graph). Peak systolic and diastolic flow in both SVC and IVC were comparable between groups. However, peak diastolic flow in the IVC occurred earlier in the cardiac cycle when corrected for heart rate in the PFO/CS group than controls (535ms v 590ms, p=0.04). The antero-posterior distance between the SVC and IVC was comparable between groups. The right-left distance between the SVC and IVC was greater in the PFO/CS group than the controls (10.6±5.3cm v 3.9±6.5cm, p=0.02)

## Conclusions

A spectrum of right atrial flow patterns can be seen in the normal heart. The relative position of the SVC and IVC may influence the generation of these. Our findings suggest that variant flow patterns in the right atrium may contribute to the pathogenesis of CS in the presence of PFO, we hypothesise, by promoting a passage for paradoxical embolism. Further work is needed to better define the subsets of CS patients in whom the PFO should be regarded as pathological.

## Funding

Academy of Medical Sciences and Newcastle Hospital Charities.

